# Three-Dimensional Dental Analysis in Subjects with Skeletal Malocclusion: A Retrospective Observational Study

**DOI:** 10.3390/dj13070280

**Published:** 2025-06-22

**Authors:** Rosanna Guarnieri, Francesca Squillace, Rachele Podda, Alfredo Salvatore Monterossi, Gabriella Galluccio, Roberto Di Giorgio, Ersilia Barbato

**Affiliations:** 1 Department of Oral and Maxillofacial Sciences, School of Dentistry, “Sapienza” University of Rome, 00185 Rome, Italy; 2 Private Practice, 87036 Cosenza, Italy

**Keywords:** 3D models, dental measurements, reliability, Bolton index, sagittal skeletal discrepancies, volumetric analysis

## Abstract

**Background:** The aim of this study was to evaluate the correlation between skeletal class and dental dimensions analyzed through linear, surface area, and volumetric measurements. **Methods:** The sample consisted of 90 patients with an average age of 18 years (44 > x > 12). The following tests were used to investigate any correlation between skeletal class and tooth size: Hoeffding’s test, Cramér’s V test, and analysis of variance (ANOVA), followed by Tukey’s post hoc HSD test and the logit model. The significance level was set at 0.050. **Results:** Cramér’s V test indicated a weak association between skeletal class (I, II, III) and total Bolton index (V = 0.167, *p* < 0.01). The ANOVA results showed that the total inferior volume and the anterior inferior volume were significantly greater (*p* = 0.012; *p* = 0.012) in skeletal class III (*p* = 0.012) than in the other two skeletal classes. The total upper surface area was significantly greater in patients with skeletal class III compared to those with classes II and I (*p* = 0.029). The anterior superior surface area was significantly greater in skeletal class III than in class II and I (*p* = 0.028). From the results of the logit analysis, it is possible to state that the third model is able to explain greater variability (21%) in terms of the distribution of results for the variables considered than the first (20%) and the second (14%). **Conclusions:** Class III skeletal malocclusions are characterized by increased tooth surface and volumetric dimensions compared to class I and class II.

## 1. Background

The prevalence of malocclusion in different age groups ranges from 20% to 93% [1,2,3,4,5]. Achieving ideal occlusion, characterized by proper molar and canine relationships, as well as optimal overjet and overbite values in harmony with facial aesthetics, requires that the teeth be proportionate to one another and in balance with the size of the dental arches and the underlying maxillary and mandibular skeletal structures.

The prevalence of tooth size discrepancies (TSDs) appears to be relatively high in the general population: 13% to 30% of patients present with clinically significant tooth size discrepancies. Additionally, other research has shown that 20% to 30% of individuals exhibit notable anterior TSDs, while overall TSDs affect approximately 5% to 14% of the population [6].

The first definition of “normal occlusion” dates back to the late 19th century. It was posited by Edward H. Angle, who proposed his own classification of malocclusions based on the positional relationship between the upper and lower permanent first molars [7]. In 1972, Andrews developed the six keys of normal occlusion (molar relationship, crown angulation, crown inclination, no rotations, tight contact points, and flat curve of Spee), a classification designed to provide a comprehensive framework for achieving ideal occlusion [8].

Although Angle’s classification is still widely used in clinical practice today, it has the major limitation of referring to malocclusion exclusively in dentoalveolar terms. A few years later, Ballard developed a classification of malocclusions based on the skeletal assessment of the sagittal relationship between the upper maxilla and the mandible [9,10].

Finally, in the 1960s Ackermann and Proffit developed a diagram that clearly illustrated the complex interconnections among the three planes of space involved in defining a facial harmony, thereby introducing the concept of aesthetics [11].

Dental assessment is a key stage in orthodontic diagnosis. In this regard, Bennett and McLaughlin expanded Andrews’s six keys of normal occlusion by introducing a seventh key: correct tooth size [12].

The Bolton index is a widely used diagnostic tool to assess tooth size relationships. It is defined as the percentage ratio between the sum of the M-D magnitudes (mm) of the lower teeth and the sum of the M-D magnitudes (mm) of the upper teeth.

Considering that dental elements erupt with their final dimensions, their macroscopic characteristics could be a valuable aid in terms of early and individualized diagnosis for the patient [13].

This topic was examined by several authors in the 20th century. Regarding prevalence, Proffit stated that “5% of the population has some degree of disproportion in size between individual teeth” [14]. Many theories have been proposed for assessing dental proportions. Amongst these, the concept of the “golden ratio”—introduced by Lombardi in the field of dentistry—is often used as a reference for achieving dental and facial aesthetic balance and harmony [15]. According to the concept of golden proportion, when viewed from the front, the lateral incisor should be 62% the width of the central incisor and the canine should be 62% the width of the lateral incisor.

Nowadays, the application of a proportional smile design enables the creation of a smile that is in harmony with facial features. Generating a visual simulation of the proposed outcome prior to initiating treatment is essential, as it may facilitate patient involvement and discussion, thereby contributing to a more satisfactory aesthetic result [16].

Almost all studies that have examined the prevalence of tooth size discrepancy (TSDs) have concluded that the use of Bolton analysis prior to orthodontic treatment is recommended and essential for treatment planning, as 20 to 30 percent of patients exhibit significant anterior TSDs and 5 to 14 percent present overall TSDs [17,18].

Space analysis plays a crucial role in mixed dentition, as it ensures proper and complete eruption of dental elements in the arch, avoiding problems such as crowding, retention, or impaction of teeth. Tooth size has long been considered an etiologic factor in spatial abnormalities, with evidence showing that discrepancies in tooth size between the maxilla and mandible can cause malocclusion [19,20]. Various methods have been proposed to assess tooth size anomalies. Amongst these, Little’s irregularity index (LII) was developed to assess tooth irregularity by measuring the linear displacements in the horizontal plane between contact points of anterior teeth. Later, Bolton analysis was developed to identify disharmony between maxillary and mandibular tooth size [20,21].

Examining dental anomalies to gain a deeper understanding of genotype–phenotype correlations may provide a new tool for the management and classification of specific malocclusions. Dimensional alterations are to be considered dental anomalies, specifically falling under the category of volume anomalies (macrodontics, microdontics, and taurism). The reported prevalence of dental anomalies differs among various studies in the literature. Some authors attribute these conflicting results to differences in ethnicity, diagnostic criteria, and environmental and nutritional factors [21,22,23,24,25,26,27]. The overall prevalence of dental anomalies is 20.09% [28]. Specifically, the most frequent anomalies comprise the displacement of the maxillary canine (7.5%), hypodontia (7.1%), and tooth impaction (3.9%).

In relation to sex, some reports suggest no statistically significant differences between males and females in the prevalence of dental anomalies [26]. Kathariya MD et al. found significant sex differences only for dental agenesis, microdontia, and accessory cusps [22]. One of the interesting results of a 2018 study by Fernandez et al. [29] is the association between microdontia and class III skeletal malocclusion. Maxillary teeth were more frequently affected by this dental abnormality, and it can be hypothesized that this finding may be attributed to maxillary deficiency, a key characteristic of class III skeletal malocclusion. Despite the association between dental anomalies and skeletal malocclusion patterns, few studies have investigated this clinical evidence [22,23,24,25,26,27].

This one of the first studies in the literature to assess dental dimensions not only in terms of linear measurements but also considering surface area and volumetric measurements [30].

The purpose of the present study was to investigate the possible correlations between skeletal class I, II, and III subjects and dental dimensions, analyzed through linear, surface area, and volumetric measurements [31,32,33,34,35].

The starting null hypothesis was that there would be no statistically significant differences in dental size among class I, II, and III subjects.

## 2. Materials and Methods

Pretreatment records of 598 Caucasian patients treated at the Department of Orthodontics of the Sapienza University of Rome between January 2021 and May 2022 were analyzed.

The following inclusion parameters were applied for sample selection: age above 12 years, permanent dentition, absence of agenesis, impacted, supernumerary and deciduous teeth, absence of gingivitis, and complete medical records, such as lateral cephalogram and plaster study models.

Exclusion parameters were incomplete medical records, craniofacial syndromes, systemic diseases, severe allergies, ongoing radiation or chemotherapy, head or neck surgery, lateral cephalograms that could not be clearly analyzed, and damaged plaster study models.

More specifically, the following inclusion criteria were used for model selection: models with complete permanent dentition, with no agenesis, impactions, gingivitis, supernumerary or deciduous teeth; structurally intact, high-quality models free of fractures or air bubbles; and models presenting either normal occlusion or dento-skeletal malocclusion confirmed by cephalometric analysis.

The patient group was not randomized. Instead, patients were selected based on the presence of specific diagnostic signs and symptoms following a predefined clinical rationale, in order to ensure the inclusion of a homogeneous and representative sample of the target population for the study or intervention.

A total of 90 subjects were selected from the original study sample.

The 90 patients were divided as follows: 30 in skeletal class I, 30 in skeletal class II, and 30 in skeletal class III, with an equal distribution of male and female sex (1:1) and a mean age of 18 years. Specifically, the sample consisted of 43 males (48%) and 47 females (52%). Among individuals with skeletal class I, 30% were male and 36% female. In skeletal classes II and III, 35% were male and 32% female.

Three-dimensional models were obtained by scanning with a Care Stream 3500 digital scanner (Smart Big © 2022 OpenTech3D, Reggiolo, Lombardy, Italy) plaster models, which were then processed using software (Meshmixer © 2020, version 3.5.474, Autodesk, Inc., San Rafael, CA, USA) to obtain linear, volumetric, and surface dental measurements. Lateral cephalograms were digitized using software (Product Name: Dental Imaging Software—Brand Name: WEBCEPH—Model Name: WEBCEPH—Software Version: 1.5.0—Date of Manufacture: 10 November 2020—FDA 510(k) Number: K220903) to acquire linear and angular skeletal measurements.

The cephalometric evaluation was conducted using Steiner’s analysis method, effective in the assessment of the skeletal and dental relationships in the sagittal plane.

To minimize bias, the operator performing the measurements was blinded to all clinical data and to the group allocation of each patient.

### 2.1. Measurements on 3-D Models

− M-D diameters of the dental elements (right first molar to left first molar) of the upper and lower arches (mm).
− Total* Bolton index (%).
− Anterior** Bolton index (%).
− Total* surface area of the clinical crowns for both the maxilla and mandible (mm^2^).
− Anterior** surface area of the clinical crowns for both the maxilla and mandible (mm^2^).
− Total* volume of the clinical crowns of the maxilla and mandible (mm^3^).
− Anterior** volume of the clinical crowns of the maxilla and mandible (mm^3^).

* Determined by the sum of the dental elements from the right first molar to the left first molar.

** Determined by the sum of the dental elements from the right first canine to the left first canine.

### 2.2. Measurements on Digital Cephalometry

− SNA: angle between the S point (sella turcica), the nasion (N), and the A point (supraspinal point of the maxilla).
− SNB: angle between the S point (sella turcica), the nasion (N), and the B point (submental point of the jaw).
− ANB: difference between the angular values of SNA and SNB.

### 2.3. Statistical Analysis

Statistical analyses were performed using Stata version 17.

The sample size was established following a power analysis performed with the GPower program, which showed that the minimum number of subjects to be included in the analyses was 88 (power = 0.80; a = 0.05; effect size = 0.30).

A single operator established the sample selection criteria. The measurements were then entered into an Excel spreadsheet and reviewed by a statistician. To verify the reliability of the initial results, a second random evaluation was conducted by the same operator after a one-month interval. The measurement error between the two assessments was calculated using Houston’s method [36]. The difference between the two observations was statistically non-significant, with an agreement rate of 98.7% for skeletal measurements and 98.5% for dental measurements.

Additionally, measurements of two randomly selected variables (i.e., width1 and upper total volume) were analyzed by a second operator after 14 days. To assess inter-operator reliability, Pearson’s correlation test and Spearman’s rank correlation test were used. Pearson’s test showed a perfect, positive, and significant correlation between the two measurements of width1 taken under different circumstances by two different operators (r = 1, *p* < 0.001). Thus, as width1 increases, width1_E also increases. Similarly, Spearman’s test applied to the variables related to the upper total volume, also measured twice by two different operators, revealed a perfect, positive, and significant correlation (*ρ* = 1, *p* < 0.001).

The difference between the two observations produced a statistically insignificant error: an equality rate of 98.7 percent was identified for skeletal measurements and 98.5 percent for dental measurements. In addition, measurements of two randomly selected variables (i.e., anterior Bolton index and overall volume) were analyzed by a second operator (R.G.) 14 days apart. Pearson’s correlation test and Spearman’s test were used to check the reliability of the inter-operator results. Pearson’s test showed a perfect positive and significant correlation between the two variables (i.e., anterior Bolton index and anterior Bolton index_E) measured under two different circumstances and by two different operators (r = 1, *p* < 0.001). Therefore, as anterior Bolton index increases, so does anterior Bolton index_E. Spearman’s test, used instead for the upper total volume variables, was also applied to two periods of time and by two different operators, and showed a positive and significant perfect correlation (*ρ* = 1, *p* < 0.001).

To test the research hypotheses, the following analyses were performed.

#### 2.3.1. Sample Description

Descriptive statistics (mean and standard deviation) were derived for all dental variables analyzed.

#### 2.3.2. Inferential Analysis

− To investigate possible correlations between skeletal class (ANB) and dental size (linear dental values, total and anterior surface values, total and anterior volumetric values, total and anterior Bolton indices), Hoeffding’s test was used. To assess potential associations between continuous variables, we employed Hoeffding’s D test, a non-parametric method capable of detecting a wide range of dependencies, including nonlinear and non-monotonic relationships. Unlike Pearson’s correlation, which assumes linearity, and Spearman’s rank correlation, which is limited to monotonic trends, Hoeffding’s test can identify more complex patterns of association without relying on specific distributional assumptions. This makes it particularly suitable for biomedical and morphometric data, where relationships among variables may not follow conventional forms. The use of Hoeffding’s test is consistent with previous studies where flexible modeling of dependence structures was required [37].
− To investigate the possible correlation between skeletal class (class I, II, III) and Bolton index (total and anterior) an additional test, Cramér’s V, was performed, as both variables are multilevel categorical [38];
− To investigate possible correlations between skeletal class (class I, II, III) and dental measurements (total and anterior surface values, total and anterior volumetric values, total and anterior Bolton indices), analysis of variance (ANOVA) was used followed by the Tukey’s post hoc HSD test [39].
− Three logit models were estimated, one for each class, to facilitate the assessment of the impact of the collected variables on the probability of belonging to a specific class. The logit model is a statistical model that relates a binary dependent variable (Y) and a set of explanatory variables (X) that are assumed to have an influence on the probability of belonging to a specific class. The model is specified as follows: classi = C + Sexi + Agei + Sup_Area_Tot_Sup + Sup_Area_Tot_Inf + Bolton_tot + Bolton_ant + Vol_tot_inf + Vol_ant_sup + εi (Where: Sexi = sex; Agei = age; Sup_Area_Tot_Sup = upper total surface area; Sup_Area_Tot_Inf = lower total surface area; Bolton_tot = total Bolton index; Bolton_ant = anterior Bolton index; Vol_tot_inf = lower total volume; Vol_ant_sup = upper anterior volume). The significance level was set at 0.050.

## 3. Results

### 3.1. Sample Description

The sample consisted of 90 patients (30 in skeletal class I, 30 in skeletal class II, 30 in skeletal class III), with a mean age of 18 years (SD 5.2). The distribution of the sample by gender is shown in Table 1.

### 3.2. Inferential Analysis

Correlations between skeletal class (ANB) and dental size (linear dental values, total and anterior surface values, total and anterior volumetric values, total and anterior Bolton indices). Hoeffding’s test [37] showed a weak nonlinear association between ANB and dental values, expressed in millimeters, of the upper-right first premolar (element 1.4, D = 0.0034, *p* < 0.05) and the lower-left lateral incisor (element 3.2, D = 0.0042, *p* < 0.05). No association was identified with the remaining tooth values. Furthermore, the same test identified a nonlinear association, also weak, between ANB and the lower (D = 0.0053, *p* < 0.05) and anterior (D = 0.0049, *p* < 0.05) total volumetric values (Table 2).

Correlation between skeletal class (class I, II, III) and Bolton index (total and anterior). Cramér’s V [40] indicated a weak association between skeletal class (I, II, III) and total Bolton index (V = 0.167, *p* < 0.01). In addition, the same index also showed a weak association between skeletal class (I, II, III) and anterior Bolton index (V = 0.13, *p* < 0.05) (Table 2).

Skeletal class (class I, II, III) and dental measurements (total and anterior surface values, total and anterior volumetric values, total and anterior Bolton indices). The results of the ANOVA test [31] showed that the total inferior volume, expressed in cubic millimeters, was significantly greater in skeletal class III (*p* = 0.012) than in the other two classes. The lower anterior volume, expressed in cube millimeters, was significantly greater in class III (*p* = 0.012) than in classes I and II.

Total upper surface area was significantly greater in patients in class III than in classes I and II (*p* = 0.029). Upper anterior surface area was significantly greater, albeit slightly, in patients in class III compared with those in classes I and II (*p* = 0.028) (Table 3).

Logit models estimated for each class. Based on the results from the logit model for class I, there were no variables (with the exception of age) that appeared to influence the probability of belonging to class I (Table 4). Each additional year of age was associated with a 25% decrease in the odds of having class I malocclusion, assuming other variables were held constant.

From the results of the logit model for class II (Table 5), the variables that seem to influence the probability of belonging to class II are: upper total surface area, total Bolton index, upper anterior volume, and anterior Bolton index.

Upper Total Surface Area: More masticatory surface area was positively associated with class II malocclusion (OR 1.05 *p* value < 0.05).

Total Bolton Index: A one-unit increase in the Bolton index was associated with a 27% decrease in odds of class II (*p* value < 0.05)

Anterior Bolton Index: A unit increase in anterior Bolton ratio was associated with a 24% increase in odds of class II malocclusion (*p* value < 0.1)

From the results of the logit model for class III, again, there are no variables (except for age) that appear to influence the probability of belonging to class III (Table 6). Each additional year of age is associated with a 22% increase in the odds of having class III malocclusion, assuming other variables are held constant.

What is worth highlighting, however, is that the third model explains a greater proportion of variability (21%) in the distribution of the outcomes among the considered variables compared to the first (20%) and second (14%) models.

## 4. Discussion

Facial and dento-maxillary characteristics may be an expression of an interaction between genetic and environmental factors that may combine to affect the growth and development of the dento-maxillofacial complex, promoting the establishment of malocclusions. Tooth size has long been considered an etiologic factor in space discrepancies, and it has been shown that a discrepancy in tooth size between the maxilla and mandible can cause malocclusion [19,20,21,40,41].

Throughout the 20th century, this topic was examined by several authors. Notably, Bennett and McLaughlin [12] introduced a seventh key to Andrews’s six keys of normal occlusion: correct tooth size.

The present research evaluated the correlation between skeletal class and dental dimensions analyzed through linear, surface area, and volumetric measurements, representing one of the first studies to investigate not only tooth dimensions expressed as linear and ratio values (Bolton index) but also as volumetric and surface values.

Ethnic origin plays a significant role in the evaluation of tooth size discrepancies, i.e., differences in mesiodistal dimensions between maxillary and mandibular teeth that can influence occlusion and smile aesthetics. The Bolton ratios, commonly used to assess the proportional relationship between the upper and lower dental arches, were originally developed based on samples of Caucasian individuals. However, these values may not be appropriate for individuals of other ethnic backgrounds, potentially leading to inaccurate diagnoses if applied universally. Understanding ethnic variability in tooth dimensions supports a more individualized and culturally sensitive diagnostic approach.

Concerning the statistical analysis conducted on the total (V = 0.167, *p* < 0.01) and anterior (V = 0.13, *p* < 0.05) Bolton index, results were consistent with the known literature, as assessed below.

Numerous studies consistently show that tooth size discrepancies are more prominent in class II and class III malocclusions, especially in the anterior segment, highlighting the potential importance of TSDs in the etiology of these malocclusion classes.

Nie Q. et al. [42] in 1999 evaluated TSD malocclusions in a sample of 300 Chinese subjects, concluding that the total ratio was greater in classes III and II, possibly representing one of the fundamental factors causing malocclusion, especially in classes II and III. Similar results were observed by O’Mahony G. et al. [43], who in 2011 analyzed the prevalence of TSDs among different malocclusions in a group of Irish subjects (240), observing that a clinically significant anterior TSD (more than two standard deviations from the Bolton mean) existed in 37.9% of the subjects. There were no differences in the prevalence of overall TSDs between the male and female groups, but most importantly, the average anterior tooth size ratio was higher in class III and class II division 2 malocclusion than in class II division 1 malocclusion and higher in class II division 2 malocclusion than in class I malocclusion. A similar study on a group of Emiratis carried out by Mohammad MG et al. [44] in 2018 considered 521 models (188 male and 333 female subjects) and concluded that there were statistically significant differences between the different malocclusion groups regarding both anterior and overall ratios; however, when comparing the malocclusion groups with Bolton’s standards, only five cases of class II malocclusion and one case of class III malocclusion had values greater than 2 SD with respect to the average Bolton values.

Consistent evidence from multiple studies shows that tooth size discrepancies are more evident in class III malocclusions. In a comparative study, Alkofide E. et al. [45] in 2002 concluded that TSDs result more frequently in class III and particularly in the anterior sector. These results align with those reported by Yang CJ’s study [46] in 2009 considering a sample of 180 subjects (60 subjects, divided into 30 male and 30 female subjects, for each type of malocclusion), in which results showed that the mean anterior and overall ratios for class III skeletal malocclusions were significantly greater than those for skeletal classes I and II. A similar outcome was documented by Fattahi HR et al. [47], who in 2006 conducted a retrospective study in an Iranian population sample evaluating the presence of TSDs in subjects with different malocclusions, concluding that the mean anterior ratio was for the entire sample statistically different from that of Bolton (77.2), and in particular how the mean anterior ratio of the class III group was greater than that of the class II group; however, there were no significant differences related to the overall ratio. The clinical outcomes observed in our study were consistent with those reported in these three studies. In the study by Yang, similar inclusion criteria were applied, and this could justify the achievement of comparable clinical outcomes.

Contrasting conclusions have been drawn in other studies published in the literature. Several authors reported no statistically significant differences between groups in their studies considering overall total. Crosby DR and Alexander CG [18] evaluated the occurrence of TSDs among different malocclusion groups in 1989 by considering a sample of 109 models and concluding how there was no statistically significant difference in the mean of ratios across groups when compared to the Bolton mean; however, larger standard deviations were observed for each group compared to the Bolton study. Similar results were reported by Al-Khateeb SN et al. [48], who in 2006 performed a comparative study in a sample of Jordanian subjects (140) aged 13 to 15 years and concluded that there were no statistically significant differences in Bolton ratios among different malocclusions. Moreover, a similar conclusion was reported by Basaran G. et al. [49] in 2006 in their study conducted on a Turkish population sample, as no significant difference was found for any ratio between the groups, and by Sercan Akyalçin et al. in their comparative study performed in 2006. Furthermore, Toshiya Endo et al. [50] evaluated the presence of TSDs among different malocclusions in a Japanese population sample (180 models) in 2008 and concluded that there were no statistically significant differences in either anterior or overall ratios among the different malocclusion groups.

Zerouaoui MF et al. [51] in 2014 evaluated variations in the Bolton index among different malocclusions in a sample (90 models) of Moroccans and concluded that there was no significant difference between the different groups of malocclusion classes [45]. Jabri MA et al. [52] in 2019 performed a review using the Medline database by selecting 66 articles in order to evaluate TSDs in different malocclusion groups. From the results obtained, it was shown that although a comparison between tooth size ratios and malocclusion groups (classes I, II, and III) was performed, many researchers did not notice significant differences.

In a recent systematic review and meta-analysis [53] on Bolton’s 2019 reports in which 53 observational studies were included, the findings show us that the aggregate estimates for the mean OI (overall index) and AI (anterior index) were 91.78% (95% confidence interval [CI] = 91.42–92.14; I^2^ = 92.87%) and 78.25% (95% CI = 77.87–78.62; I^2^ = 90.67%), respectively. The findings of this meta-analysis show that the mean values of OI and AI differed from the original Bolton values, sex had almost no impact on mesiodistal tooth proportion, mesiodistal tooth size was proportionally larger in class II division 2 only for mean values of OI and in class III for both mean values of OI and AI.

Together, these studies underscore the significant role that tooth size discrepancies play in the development of class II and class III malocclusions, with a notable impact on anterior tooth dimensions.

Indeed, it is possible to state from the results of our analysis that there is a correlation between the variable malocclusion and the variables total Bolton index and anterior Bolton index, but it is not possible to establish the nature of this correlation. In addition, the presence of significantly increased volumetric and dental surface values in class III subjects compared to class I and II subjects makes one speculate about a possible “harmonic” increase in tooth size that would justify an almost unchanged Bolton index in spite of different, specifically increased, Volumetric and/or Surface tooth sizes.

What is certainly apparent is that there is greater variability in skeletal class III (Pseudo r-squared 0.212) in terms of tooth size than in class II (Pseudo r-squared 0.143), and given the etiopathogenetic basis of such malocclusion, one might think of a common genetic basis of phenotypic alteration, assuming that teeth and bones are offspring of different embryological pathways.

Is there, then, the possibility that the III skeletal classes are underlain not only from a skeletal point of view but also from a dental point of view by genetic alterations?

Several studies in the literature have identified locus and genes strongly associated with mandibular prognathism, supporting the multifactorial etiology of such malocclusions. In particular, Atteeri A. et al. [54] demonstrated how the rs10850110 polymorphism of the MYO1H gene has a statistically significant association with mandibular prognathism, as the G allele of the rs marker was overrepresented compared to the A ally in cases of mandibular prognathism, leading to the conclusion that the MYO1H gene is associated with an increased risk of mandibular prognathism. Furthermore, Tassopoulou-Fishell M. et al. [55] studied a North American family, identifying five regions related to mandibular prognathism: 1p22.1, 3q26.2, 11q22, 12q13.13 and 12q23. From the study of these regions, an association with MYO1H was found in the 12q23 region in North American subjects. Specifically in this study, genes coding for skeletal muscle alpha-actin ACTN2 (which is expressed in all muscle fibers) and ACTN3 (which is expressed in fast-twitch fibers) were tested in two groups of people, marathon runners who run long distances and sprint runners. From the study conducted, a particular R577X mutation was found to be increased in people who ran long distances and reduced in sprint runners. It was found that individuals with skeletal class II more frequently presented two copies of the R577X mutation and fewer fast-twitch muscle fibers in the masseter, suggesting how muscle tissue plays a key role in the establishment and severity of skeletal deformities. From a preliminary whole-genome association study [56] (GWAS) regarding mandibular prognathism, two loci (1q32.2 and 1p22.3) were found to be susceptible to mandibular prognathism and two genes were identified as candidates, PLXNA2 and SSXI2P.

Regarding genes and pathways associated with sagittal class II skeletal malocclusions, a systematic review [57] reanalyzed whole-genome association studies (GWASs), highlighting the most highly expressed genes in the third classes: MATN1 (matrilin 1), COL2A1 (collagen type II alpha 1 chain), FGFR2 (fibroblast growth factor receptor), KAT6B (lysine acetyltransferase), MYO1H (myosin IH), PLXNA2 (plexin A2), and SSX2IP (and SSX family member 2-interacting protein). In that study, 19 genes associated with class II skeletal malocclusions and 53 genes associated with class III skeletal malocclusions were found. Most of these genes correlated with cartilage and bone growth and regulation.

In cases of malocclusion, the evaluation of dental developmental anomalies may enhance the understanding of genotype–phenotype correlations, providing a useful tool for the management and categorization of specific clinical conditions [58].

Different frequencies for dental anomalies are reported in the literature. Some authors attribute these conflicting results to differences in ethnicity, diagnostic criteria, and environmental and nutritional factors [22,23,24,25,26,27,59].

Thongudomporn U and Freer TJ observed a 74.7% prevalence of dental anomalies in 111 orthodontic patients [27]. Uslu et al. reported a 40.3% prevalence of dental anomalies among orthodontic patients, with dental agenesis, evagination, and invagination as the most common tooth anomalies [24]. In relation to sex, some reports suggest no statistically significant differences between males and females in the prevalence of dental anomalies. Kathariya MD et al. found significant differences between the sexes only for dental agenesis, microdontia, and accessory cusps [22]. One of the interesting findings of a 2018 study by Clarissa Christina Avelar Fernandez et al. is the association between microdontia and class III skeletal malocclusion. This dental abnormality was more frequently observed in maxillary teeth, a finding that may be explained by the presence of maxillary deficiency, a characteristic feature of class III skeletal malocclusion [29].

Despite the association between dental anomalies and skeletal malocclusion patterns, few studies have investigated this clinical evidence [60,61].

Is it possible to speak of macrodontia? Is it possible that there is a link between malocclusion and dental abnormalities?

While the study provides valuable insights, several limitations should be taken into account. Firstly, discussing macrodontia is never simple, because there are no standardized reference parameters in the literature. In fact, the clinical interpretation of higher volumetric values in class III subjects must be discussed with greater caution, because it could actually be “macrodontia” or a normal variation within skeletal patterns.

Furthermore, the hypothesis of a harmonious relationship between volume and surface area could be influenced by the morphology of the crown or the resolution of the scan. This aspect must be considered as a possible limitation of the results obtained in the study, which therefore needs to be validated by further investigations.

Regarding volume and surface area, no study was found in the literature to allow comparison of the results obtained. A significant strength of this research is its novel approach to addressing not only dental dimensions expressed as linear and ratio values (Bolton index) but also as volumetric and surface area values. Hence, this is one of the first studies to evaluate the association of dental discrepancies (assessing individual elements in the three spatial dimensions) with different skeletal malocclusions.

It would be advisable to conduct future studies focused on defining phenotype–phenotype correlations. Phenotypes are the result of gene expression, the influence of environmental factors, and their possible interaction. These elements are essential for successful genetic studies that depend on a well-characterized phenotype. In addition to the present study, previous research has supported the hypothesis that the same genes and/or pathways may contribute to some types of dental abnormalities and skeletal malocclusions. However, a genetic study to associate them has not yet been performed [25,26,27,28,29,30,31,32,33,34,35,36].

Further and more in-depth studies are required. The findings of this study may lay the foundation for the exploration of new research horizons. Is there a possibility of building databases to categorize skeletal and dental phenotypes into dysgnathic patterns, thereby facilitating more timely diagnoses and enhancing the reliability of treatment plans? Is it possible that in class III there is a common genetic basis characterizing not only the skeletal pattern, as already known, but also the dental phenotype?

Although the data collected in this study were not designed to directly demonstrate a cause-and-effect relationship between genetic factors and changes in tooth size, the work nevertheless has exploratory and theoretical value. It may indeed stimulate new hypotheses and guide future studies, which could use advanced bioinformatic methods to better understand the genetic origins of malocclusions.

Hence, this work does not establish causal links between genetics and dental malformations, but opens the door to future studies oriented towards the use of bioinformatics to investigate the genetic origin of malocclusions in a more consistent and systematic manner.

## 5. Conclusions

When compared to class I and class II dento-skeletal malocclusions, class III dento-skeletal malocclusions are characterized by increased surface and volumetric tooth dimensions.

Dental analysis of linear, surface, and volumetric dimensions is recommended to correctly plan diagnosis and treatment.

## Figures and Tables

**Table 1:** Distribution of the sample by gender.

	n	%M	%F
Sample	90	48	52
Class I	30	30	36
Class II	30	35	32
Class III	30	35	32

**Table 2:** Descriptive statistics of the ANB angles, dental diameters, surface area, volume and Bolton Index.

Variable	Obs	Mean	Std.Dev.	Min	Max
ANB	90	2.1007	4.2879	−9.17	11
D15	90	7.0765	0.4876	5.557	8.587
D16	90	10.8231	0.7093	8.25	12.538
D14	90	7.2395	0.557	5.792	8.728
D13	90	8.0876	0.6373	6.025	9.636
D12	90	7.2024	0.5592	5.626	8.757
D11	90	8.9318	0.6448	7.115	10.541
D21	90	8.9676	0.6335	6.98	11.017
D22	90	7.0042	0.5544	5.766	8.874
D23	90	8.0258	0.6277	6.161	9.646
D24	90	7.277	0.5785	5.351	9.221
D25	90	7.0436	0.4952	5.649	8.366
D26	90	10.8232	0.7202	8.998	13.463
D36	90	11.1984	0.6716	8.353	12.677
D35	90	7.6295	0.4535	6.543	9.288
D34	90	7.3783	0.4923	6.312	8.765
D33	90	7.1531	0.6259	5.383	8.484
D32	90	6.1791	0.4992	4.948	7.508
D41	90	5.6335	0.4265	4.544	7.142
D31	90	5.6096	0.3922	4.555	6.714
D42	90	6.2095	0.5225	4.959	7.833
D43	90	7.8896	0.8466	5.734	7282
D44	90	7.3814	0.4892	6.118	8.555
D45	90	7.5627	0.5662	6.183	9.117
D46	90	11.1991	0.8182	9.145	13.758
Surface area Tot Sup	90	2440.672	344.5682	1440.01	3254.23
Surface area Tot Inf	90	2040.161	284.6851	1315.65	2661.82
Surface area ant sup	90	1171.523	165.3927	691.2048	1562.03
Surface area ant inf	90	775.2613	108.1803	499.947	1011.492
Vol Tot Sup mm^3^	90	2288.58	566.4049	1176.57	3624.61
Vol Tot Inf mm^3^	90	1774.072	442.7722	972.34	3053.49
Vol Ant Sup mm^3^	90	1098.519	271.8743	564.7536	1739.813
Vol Ant Inf mm^3^	90	674.1474	168.2535	369.4892	1160.326
Bolton index Tot	90	171.644	760.667	85.7055	7307.75
Bolton index Ant	90	241.8613	1549.662	71.2142	14,800

Abbreviations: D, mesiodistal diameter, Max, maximum; Min, minimum; Obs, remarks; Std.Dev., standard deviation; Vol, volume; Tot, total; Sup, superior; Inf, inferior; Ant, anterior.

**Table 3:** Results of ANOVA and Tukey’s post hoc HSD sensitivity test.

Parameters	Class I (n = 30)		Class II (n = 30)		Class III (n = 30)		ANOVA *p*-Value	Tukey’s Post Hoc HSD Test		
	Median	±SD	Median	±SD	Median	±SD		I and II *p*-Value	I and III *p*-Value	II and III *p*-Value
Upper total volume (mm^3^)	2157.6	521.5	2304	631.1	2404.1	531.5	0.239	0.575	0.213	0.771
Lower total volume (mm^3^)	1602.3	411.8	1702.2	443.9	1937.7	420.3	0.012 *	0.236	0.008 **	0.337
Upper anterior volume (mm^3^)	1035.6	250.3	1105.9	302.9	1153.96	255.1	0.240	0.580	0.215	0.781
Anterior inferior volume (mm^3^)	608.9	156.5	677.2	168.7	736.3	159.7	0.012 *	0.245	0.009 **	0.354
Upper total surface area (mm^2^)	2305.8	325.4	2507.4	355	2508.8	322.8	0.029 *	0.056	0.054	1.000
Lower total surface area (mm^2^)	1944.6	227.1	2073.3	258.3	2102.6	340.3	0.071	0.181	0.078	0.913
Upper anterior surface area (mm^2^)	1106.8	156.2	1203.6	170.4	1204.2	154.9	0.028 *	0.061	0.076	1.000
Lower anterior surface area (mm^2^)	738.9	86.3	787.8	98.1	799.1	129.3	0.072	0.181	0.078	0.913
Total Bolton index	91.3	2.2	331.6	1317.6	92	2.6	0.374	0.443	1.000	0.445
Anterior Bolton index	78.2	3.3	569	2684	78.4	2.4	0.371	0.441	1.000	0.441

** *p* < 0.05, * *p* < 0.1.

**Table 4:** Logistic regression, class I.

Class I	Coef.		St.Err.	t-Value	*p*-Value	[95% Conf		Interval]	Sig
Age	−0.286		0.085	−3.36	0.001	−0.452		−0.119	***
Sex	0.238		0.540	0.44	0.659	−0.820		1.296	
Lower Tot Vol	0.000		0.001	0.27	0.784	−0.002		0.003	
Tot Surf Are	−0.001		0.001	−1.28	0.199	−0.004		0.001	
Tot Surf Are Ant	−0.001		0.002	−0.61	0.540	−0.004		0.002	
Constant	8.940		3.221	2.77	0.006	2.627		15.254	***
Mean dependent var		0.333		SD dependent var			0.474		
Pseudo r-squared		0.20		Number of obs			90.000		
Chi-squared		24.802		Prob > chi2			0.000		
Akaike crit. (AIC)		101.771		Bayesian crit. (BIC)			116.770		

Abbreviations: Surf, surface; Are, area; Vol, volume; Tot, total; Ant, anterior. *** *p* < 0.01.

**Table 5:** Logistic regression, class II.

Class II	Coef.		St.Err.	t-Value	*p*-Value	[95% Conf		Interval]	Sig
Age	−0.005		0.049	−0.10	0.924	−0.101		0.092	
Sex	0.304		0.537	0.57	0.572	−0.748		1.355	
Lower Tot									
Vol	0.000		0.001	−0.25	0.800	−0.003		0.002	
Tot Surf									
Are	0.005		0.002	2.21	0.027	0.001		0.010	**
Tot Surf									
Are	0.001		0.002	0.82	0.415	−0.002		0.005	
Tot Bolton									
Ind	−0.313		0.152	−2.06	0.039	−0.610		−0.015	**
Upper Ant									
Vol	−0.006		0.003	−2.06	0.039	−0.012		0.000	**
Ant Bolton									
Ind	0.212		0.116	1.83	0.067	−0.015		0.439	*
Constant	2.683		11.726	0.23	0.819	−20.299		25.665	
Mean dependent var		0.333		SD dependent var			0.474		
Pseudo r-squared		0.143		Number of obs			90.000		
Chi-squared		16.341		Prob > chi2			0.038		
Akaike crit. (AIC)		116.232		Bayesian crit. (BIC)			138.730		

Abbreviations: Surf, surface; Are, area; Vol, volume; Tot, total; Ant, anterior; Ind, index. ** *p* < 0.05, * *p* < 0.1.

**Table 6:** Logistic regression, class III.

Class III	Coef.		St.Err.	t-Value	*p*-Value	[95% Conf		Interval]	Sig
Age	0.195		0.068	2.88	0.004	0.063		0.328	***
Sex	−0.483		0.569	−0.85	0.396	−1.599		0.632	
Lower Tot									
Vol	0.002		0.001	1.35	0.178	−0.001		0.004	
Tot Surf									
Are	0.001		0.002	0.56	0.574	−0.003		0.005	
Tot Surf									
Are	−0.001		0.002	−0.86	0.390	−0.004		0.002	
Tot Bolton									
Ind	0.173		0.144	1.20	0.229	−0.109		0.454	
Upper Ant									
Vol	−0.002		0.003	−0.70	0.483	−0.007		0.003	
Ant Bolton									
Ind	−0.132		0.129	−1.03	0.305	−0.385		0.120	
Constant	−10.556		11.329	−0.93	0.351	−32.760		11.648	
Mean dependent var		0.333		SD dependent var			0.474		
Pseudo r-squared		0.212		Number of obs			90.000		
Chi-squared		24.282		Prob > chi2			0.002		
Akaike crit. (AIC)		108.290		Bayesian crit. (BIC)			130.789		

Abbreviations: Surf, surface; Are, area; Vol, volume; Tot, total; Ant, anterior; Ind, index. *** *p* < 0.01.

## Data Availability

The datasets used and/or analyzed during the current study available from the corresponding author on reasonable request.
